# Modulations of Heart Rate, ECG, and Cardio-Respiratory Coupling Observed in Polysomnography

**DOI:** 10.3389/fphys.2016.00460

**Published:** 2016-10-25

**Authors:** Thomas Penzel, Jan W. Kantelhardt, Ronny P. Bartsch, Maik Riedl, Jan F. Kraemer, Niels Wessel, Carmen Garcia, Martin Glos, Ingo Fietze, Christoph Schöbel

**Affiliations:** ^1^Interdisziplinäres Schlafmedizinisches Zentrum, Charité – Universitätsmedizin BerlinBerlin, Germany; ^2^International Clinical Research Center, St. Anne's University Hospital BrnoBrno, Czech Republic; ^3^Naturwissenschaftliche Fakultät II – Chemie, Physik und Mathematik, Institut für Physik, Martin-Luther Universität Halle-WittenbergHalle, Germany; ^4^Kardiovaskuläre Physik, Arbeitsgruppe Nichtlineare Dynamik, Fachbereich Physik, Humboldt-Universität BerlinBerlin, Germany; ^5^Physics Department, Bar-Ilan-UniversityRamat Gan, Israel

**Keywords:** sleep stages, autonomic function, heart rate, ECG, cardiovascular regulation, sleep apnea

## Abstract

The cardiac component of cardio-respiratory polysomnography is covered by ECG and heart rate recordings. However, their evaluation is often underrepresented in summarizing reports. As complements to EEG, EOG, and EMG, these signals provide diagnostic information for autonomic nervous activity during sleep. This review presents major methodological developments in sleep research regarding heart rate, ECG, and cardio-respiratory couplings in a chronological (historical) sequence. It presents physiological and pathophysiological insights related to sleep medicine obtained by new technical developments. Recorded nocturnal ECG facilitates conventional heart rate variability (HRV) analysis, studies of cyclical variations of heart rate, and analysis of ECG waveform. In healthy adults, the autonomous nervous system is regulated in totally different ways during wakefulness, slow-wave sleep, and REM sleep. Analysis of beat-to-beat heart-rate variations with statistical methods enables us to estimate sleep stages based on the differences in autonomic nervous system regulation. Furthermore, up to some degree, it is possible to track transitions from wakefulness to sleep by analysis of heart-rate variations. ECG and heart rate analysis allow assessment of selected sleep disorders as well. Sleep disordered breathing can be detected reliably by studying cyclical variation of heart rate combined with respiration-modulated changes in ECG morphology (amplitude of R wave and T wave).

## Introduction

Polysomnography primarily focuses on the recording of neurophysiological signals derived from the skull, such as electroencephalography (EEG), electrooculography (EOG), and electromyography (EMG). EEG electrodes are placed on well-defined head positions according to the 10–20 system (Berry et al., 2014). Sleep follows a sequence of sleep stages with light sleep, deep sleep, and REM sleep in repetitive cycles through normal sleep in healthy adults. Metabolic, immune, and autonomous nervous system functions show varying activity closely following these sleep stages.

In order to investigate the autonomous nervous system functions during sleep, respiration, body movement, electrocardiography (ECG), and—quite recently—blood pressure are registered as part of cardiorespiratory polysomnography (Berry et al., 2014; Roebuck et al., 2014). This review focuses on ECG and ECG-derived heart rate during sleep. According to current recommendation, only one channel of ECG is usually recorded. Hence, cardiovascular regulation is unfortunately often underrepresented in cardiorespiratory polysomnography. Only more recent polysomnography systems offer a new way to calculate non-invasive blood pressure from recordings of a photoplethysmographically derived pulse wave on the finger (Gesche et al., 2012). The pulse wave is easily obtained from regular oxygen saturation sensors, if configured appropriately. Together with the ECG, it allows the calculation of pulse transit time (PTT). PTT can be used as a surrogate for blood pressure, adding an important component to cardiovascular monitoring during sleep. Although quantitative continuous recordings of blood pressure by, e.g., Portapres would be more accurate, they are very expensive, not trivial to apply, and as a consequence not much used in sleep studies. Blood pressure recordings are not discussed in this review.

Heart rate fluctuations are calculated from the recording of a single channel ECG. For sleep studies this is sufficient. Tracking of nocturnal variations of the sympathetic and parasympathetic nervous system functions is possible. Additionally an ECG during attended cardiorespiratory polysomnography in a sleep laboratory or at other clinical services (pneumology, neurology, anesthesiology, intensive care unit) serves as an online monitor for vital functions during a sleep study (Penzel et al., 1993). A single channel ECG is sensitive enough to detect bradycardia, tachycardia, arrhythmias, paroxysmal atrial fibrillation, and AV block. Occasionally a single channel recording indicates nocturnal coronary ischemia (Caples et al., 2007). ECG recording enables initial evaluation of nocturnal arrhythmia in terms of heart-rate variability (HRV) and ectopic beats (Caples et al., 2007). This can be sufficient when a differential diagnosis of cardiac arrhythmia is not needed. The reduced recording can support the indication for a more comprehensive cardiac examination by multi-channel ECG recording or by long-term ECG recording with three or more ECG leads.

During sleep, the autonomic nervous system is subject to pronounced changes and variability (Snyder et al., 1964). These alterations are linked to sleep stages, and differ between sleep stages profoundly. This is so impressive that sleep itself had been called a “challenging test for the autonomic nervous system” (Verrier et al., 1996). We have learned from basic research studies in animals and humans that the autonomic nervous system activity changes in a characteristic way with the sleep stages (Somers et al., 1993; Trinder et al., 2001). Seminal investigations that recorded muscle sympathetic nerve activity (MSNA) have documented a profound attenuation in neuron firing activity. This progresses from wakefulness, throughout light sleep, and to deep sleep. The lowest activity of the sympathetic nervous system during deep sleep/slow wave sleep—sleep stage N3—is associated with predominant activity of the parasympathetic nervous system. During rapid eye movement sleep (REM sleep) an increased MSNA had been found again. This REM-sleep associated activity appears to be irregular, it occurs in bursts. This contrasts to high MSNA activity during wakefulness, which is correlated with physical and mental workloads.

Analysis of fluctuations in heart rate has been performed by classic techniques of calculating HRV with frequency analysis using spectral analysis (Akselrod et al., 1981). Fourier analysis had been applied first. Over the last decades, spectral analysis of heart rate proved to be successful because frequency components were related to autonomous nervous system components (Tobaldini et al., 2013). Other spectral analysis methods beside Fourier analysis were applied. Spectral analysis had been widely accepted because physiological functions were attributed to frequency components. The low frequency content of HRV (LF, 0.04–0.15 Hz) reflects both sympathetic and vagal modulations. The high-frequency range (HF, 0.15–0.4 Hz) is associated with respiration and reflects the activity of the parasympathetic nervous system (Task force of the European Society of Cardiology and the North American Society of Pacing and Electrophysiology, 1996). Discussions on the physiological significance of very low frequencies (VLF, below 0.04 Hz) are still ongoing. Sleep-related breathing disorders, vasomotor activities, and thermoregulation may contribute to these VLF components. These relationships are not fully explored. However, other effects may contribute to the VLF components. If a subject has a low breathing frequency, e.g., breathing at a rate of 9–10 breaths per minute during sleep, then the spectral power is shifted toward lower frequencies. This will result in impaired LF and HF components and all subsequent spectral analysis values are no longer interpretable.

Considering the physical signal amplitude of ~1 mV, the ECG is the largest electrophysiological signal on the human body surface. When digitally recorded the signal is digitized with at least 100 Hz to preserve the waveform. In more recent equipment, and according to guidelines, ECG should be digitized with 500 Hz (Berry et al., 2014). The technical recommendation is the same as used for digitization of the electrophysiological parameters from the skull. ECG digitization below 500 Hz is associated with limitations involving the detection of small heart rate variations and distinct waveform patterns. With respect to amplifier technology, and compared to EEG, EOG, and EMG, ECG signals are simple to record due to their large signal amplitudes. Signal to noise ratio is much better and requires less sophisticated amplifiers. Consequently, and based on knowledge of the underlying physiology of the autonomic nervous system, ECG became a highlighted candidate for developing a simple tool for studying sleep and diagnosing sleep disorders (Penzel et al., 2015). The ECG and derived calculations may serve as a simple surrogate parameter for sleep recording on the skull with EEG, EOG, and EMG.

Here we describe developments and possibilities, as well as the present status of limitations of this future directing approach: recording sleep stages and sleep disorders from a single channel ECG. This approach is on the edge of reducing sleep studies to one directly (with sensors on the body) recorded physiological signal. This recording offers a high diagnostic value in terms of cardiac screening as well. Regarding our selection and presentation of studies reviewed here, it has been possible to consider only a restricted number of studies. The selection of approaches and algorithms reflects our personal group perspective in a chronological order and may not reflect all possible approaches. With this specific approach our review complements an earlier review on HRV in normal and pathological sleep which was organized according to methods applicable and to a selection of selected sleep disorders (Tobaldini et al., 2013).

## Effects of sleep stages and sleep apnea on heart rate variability

### Cyclical variation of heart rate with sleep apnea

**Figure 1:**
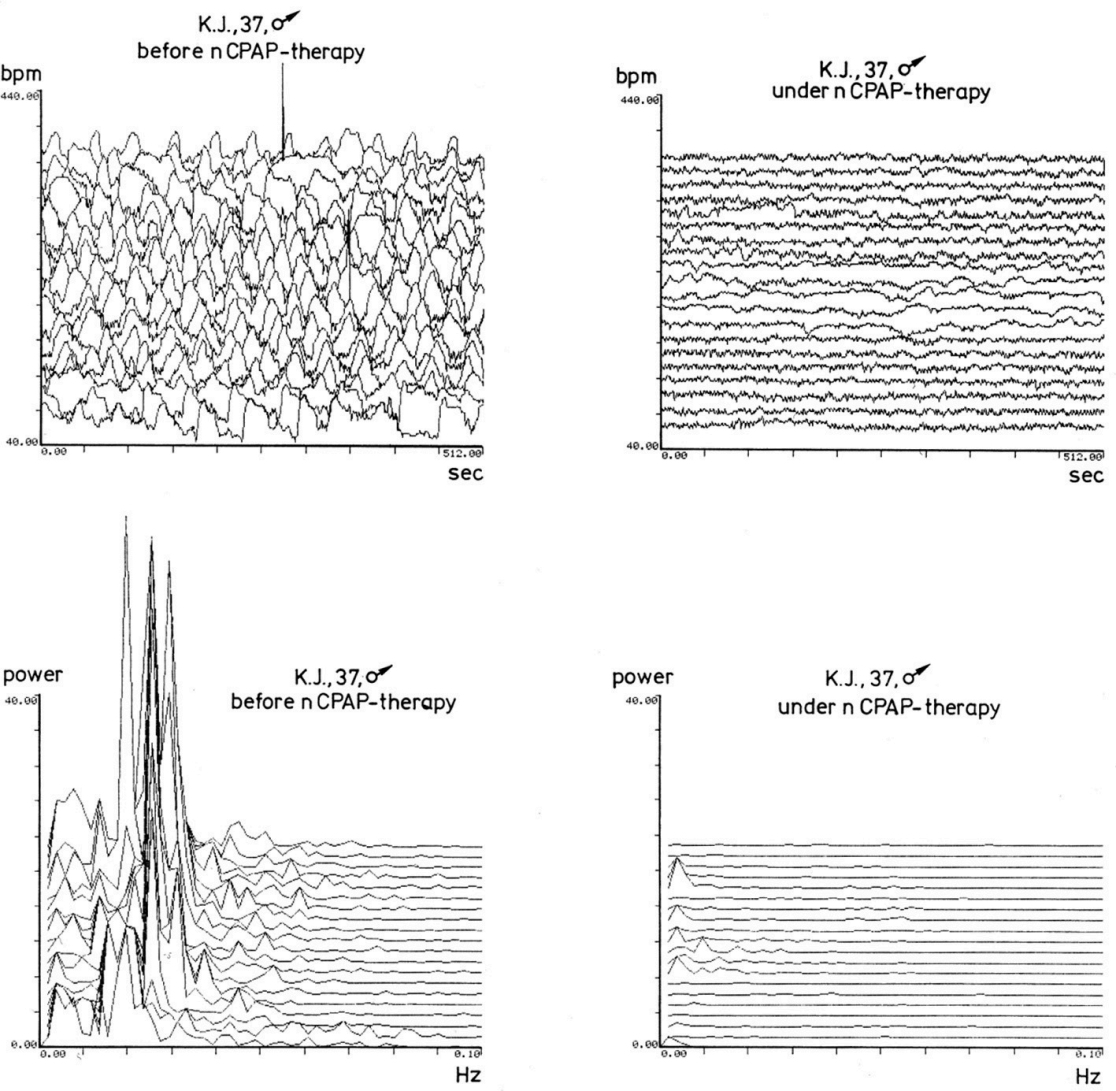
**This figure shows cyclical fluctuations of heart rate, in beats per minute, for a patient with obstructive sleep apnea, before CPAP therapy, in the time window at the top left**. The plot shows 512 s of nocturnal heart rate per line. Below this plot is the spectral analysis of this same time window. To the right, in the same type of presentation, is the heart rate of the same patient after initiation of CPAP therapy. The cyclical fluctuations of heart rate that occur during sleep apnea have been eliminated. Lower, at the bottom right, is again the spectral analysis of the heart rate. The low frequencies, characteristic for apnea—around 0.02 Hz of the heart rate variability—have disappeared. This figure originated in the Sleep Lab of the University of Marburg, Germany, and was created around 1990 from digital recordings by an Atari computer.

A few early reports observed and described the respiratory pattern of sleep apnea (Burwell et al., 1956). But these early reports could not show all physiological conditions linked to this pathology. Guilleminault described obstructive sleep apnea and its collapse of the upper airways during sleep for 10 to typically 60 s, occurring several 100 times per night, as a new pathological entity. He observed and described characteristic variations in heart rate observed in parallel to obstructive apnea events (Guilleminault et al., 1984). This pattern can be observed best when plotting heart rate as a beat-to-beat “tachogram.” Plotting heart rate in this way is the first procedure to be performed before any sophisticated analysis algorithms for heart rate processing are applied (Stein et al., 2003; see Figure 1 upper part). The phenomenon observed in parallel to apnea events was described as cyclical variation in heart rate by Guilleminault. The study by Guilleminault proposed to use this characteristic pattern for diagnosing sleep apnea (Guilleminault et al., 1984). Many diagnostic devices—intended to detect and diagnose sleep apnea outside of a sleep laboratory, so called out-of-center sleep recordings, in fact made use of this heart-rate pattern just by plotting the beat-to-beat tachogram (Penzel et al., 1990; Collop et al., 2011).

Influences of snoring and respiratory timing on heart rate as a reflection for sympathetic and parasympathetic activity were investigated further. With onset of snoring an increase in inspiratory time (Ti) and expiratory time (Te) is observed (Stoohs and Guilleminault, 1991). With continuous snoring, Ti increases further and Te decreases. These research results did trigger investigations on respiratory motor output and MSNA (St. Croix et al., 1999). It could be shown, that MSNA was maximal at end-expiration and minimal at end-inspiration. This demonstrates an additional influence of lung inflation feedback on sympathetic discharge and suggests that feedback from baroreceptors and pulmonary stretch receptors are dominant determinants of the respiratory modulation of MSNA (St. Croix et al., 1999). Fatiguing inspiratory muscles, as observed with prolonged inspiration, can increase MSNA even more (St. Croix et al., 2000). These physiological mechanisms are mentioned but not further considered in this review.

During each apnea event, relative bradycardia becomes apparent. Due to the cessation of breathing, the level of oxygen in the blood decreases. This is monitored best by partial oxygen pressure (pO_2_). Because it is difficult and expensive to monitor pO_2_ non-invasively and continuously, sleep laboratories monitor oxygen saturation (SaO_2_) using pulse oximetry on the finger (SpO_2_). It must be kept in mind that due to the physiological oxygen binding curve oxygen saturation (SaO_2_) drops much slower than partial oxygen pressure at high values. Furthermore, most often oxygen saturation is recorded on the finger (SpO_2_), which is the periphery of the body, to which a lower blood oxygen content needs to travel (circulatory delay). With these physiological considerations, oxygen desaturation events on the finger (SpO_2_) are often delayed compared to apnea events recorded by respiratory sensors directly. Furthermore, desaturation events are often not as severe as expected, depending on baseline oxygen partial pressure and the impact of the oxygen binding curve. The drop in heart rate is a physiological reaction to the apnea event in order to improve blood gas exchange in the lung when not breathing, most likely mediated by parasympathetic activity. This reflex is known as the “diving reflex” observed in healthy persons as well. The reduction in heart rate (diving reflex) is followed by acceleration in heart rate, a relative tachycardia, which promotes blood gas exchange in the lungs after the apnea event, when the upper airway occlusion ends, the airways re-open and subsequent, increased breathing occurs. Today we assume that the very rapid increase in heart rate at the end of apnea is not due to an additional increase in MSNA, which is already high and is increasing during the apnea event, but it is due to an immediate cessation of parasympathetic activity at the end of apnea. We assume that parasympathetic activity is also high during the apnea event and ceases with the onset of respiration at the end of each apnea event. Thereby MSNA becomes the major component causing an increase in heart rate and blood pressure, both being further regulated by baroreceptor reflex.

Superimposed in addition, a decline of mean heart rate is observed over the time course of the night (Snyder et al., 1964). This decrease is another fundamental physiological mechanism during normal sleep, described in more detail below.

These patterns taken together, arising from alterations in sympathetic nervous activity and parasympathetic nerve activity during each apnea event provide a heart rate pattern which is very typical for sleep apnea, just like a signature. As a result, it is possible to count the number of apnea events by counting the signature patterns of cyclical HRV. The MESAM system and its successors, as well as new polygraph devices, employ a simple plotting of heart rate for diagnosis of sleep apnea in an outpatient context (Penzel et al., 1990; Roos et al., 1993; Stein et al., 2003).

**Figure 2:**
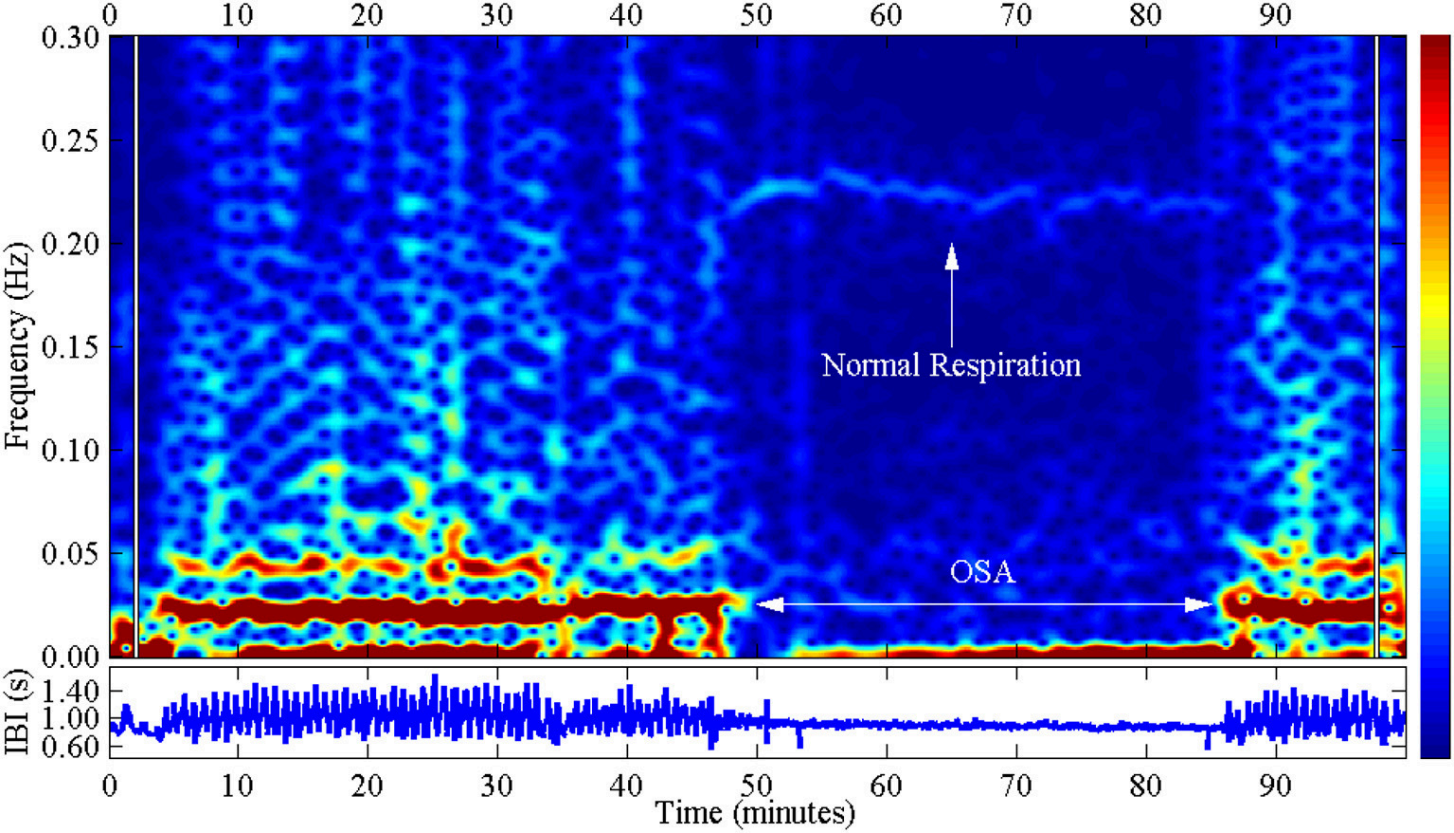
**This figure shows the spectral analysis of the cyclical variation in heart rate with obstructive sleep apnea (OSA): left and right**. This is evident in the red zones at frequencies between 0 and 0.04 Hz, which belong to the very low frequency (VLF) band. The central area shows only normal respiration, evidenced by the bright strip in the frequency range around 0.2 Hz, which belongs to the high frequency (HF) band. IBI stands for “interbeat interval” and shows the inverse heart rate. This figure originated at the Computers in Cardiology Competition (taken from Penzel et al., 2002).

Since the cyclical variation in heart rate presents such a prominent periodic pattern, it was logical to apply mathematical methods of frequency analysis and spectral analysis to quantitatively assess the cyclical variation in heart rate (Figure 1 lower part). The initial aim of this analysis was to determine the degree of severity of sleep-related breathing disorders. Various teams have employed the method of Fourier analysis—which has successfully been applied for other aspects of sleep analysis, specifically sleep EEG analysis—for identifying rhythmic activities as well (Figure 2). Limitations subsequently arose in the attempt to evaluate automatically the periodic pattern by use of Fourier analysis (Ivanov et al., 1996). In general, the cyclical variations are not as uniformly periodic as seen in well selected patients. Apnea and hypopnea event durations are not constant, with a large variation in duration of apnea events, especially associated with the different sleep stages. In addition a superimposed variability of heart rate which accompanies sleep stages and REM sleep in particular (Somers et al., 1993), is observed as well. And furthermore, the longer a patient suffers from undiagnosed obstructive sleep apnea, the less the physiological compensating diving reflex may protect him and the dipping of heart rate during the apnea event becomes less and gets more impaired (Stein et al., 2003).

The application of period analysis and spectral analysis has many limitations. The amplitude of cyclical variation of heart rate (difference between minimum heart rate during apnea event und maximum heart rate during compensating paced breathing) depends on physical training condition, age, weight, and concomitant diseases. For example diabetes, which impairs autonomic function and heart rate fluctuations, obviously reduces the amplitude of cyclical variation of heart rate. Often an individual characteristic pattern of bradycardia and tachycardia, like a personal signature is found. This is further influenced by concomitant cardiologic diseases, arrhythmia, pacemaker ECGs, and heart failure—which make the signature difficult to interpret. Therefore, automatic assessment of sleep-related breathing disorders based on cyclical heart-rate variation alone, is very difficult to achieve (Penzel et al., 2015). For home sleep testing of sleep apnea the determination of oxygen saturation and more direct recording of breathing disorders are simpler and essential for greater diagnostic reliability (Roos et al., 1993).

### Regulation of heart rate in the various sleep stages

**Figure 3:**
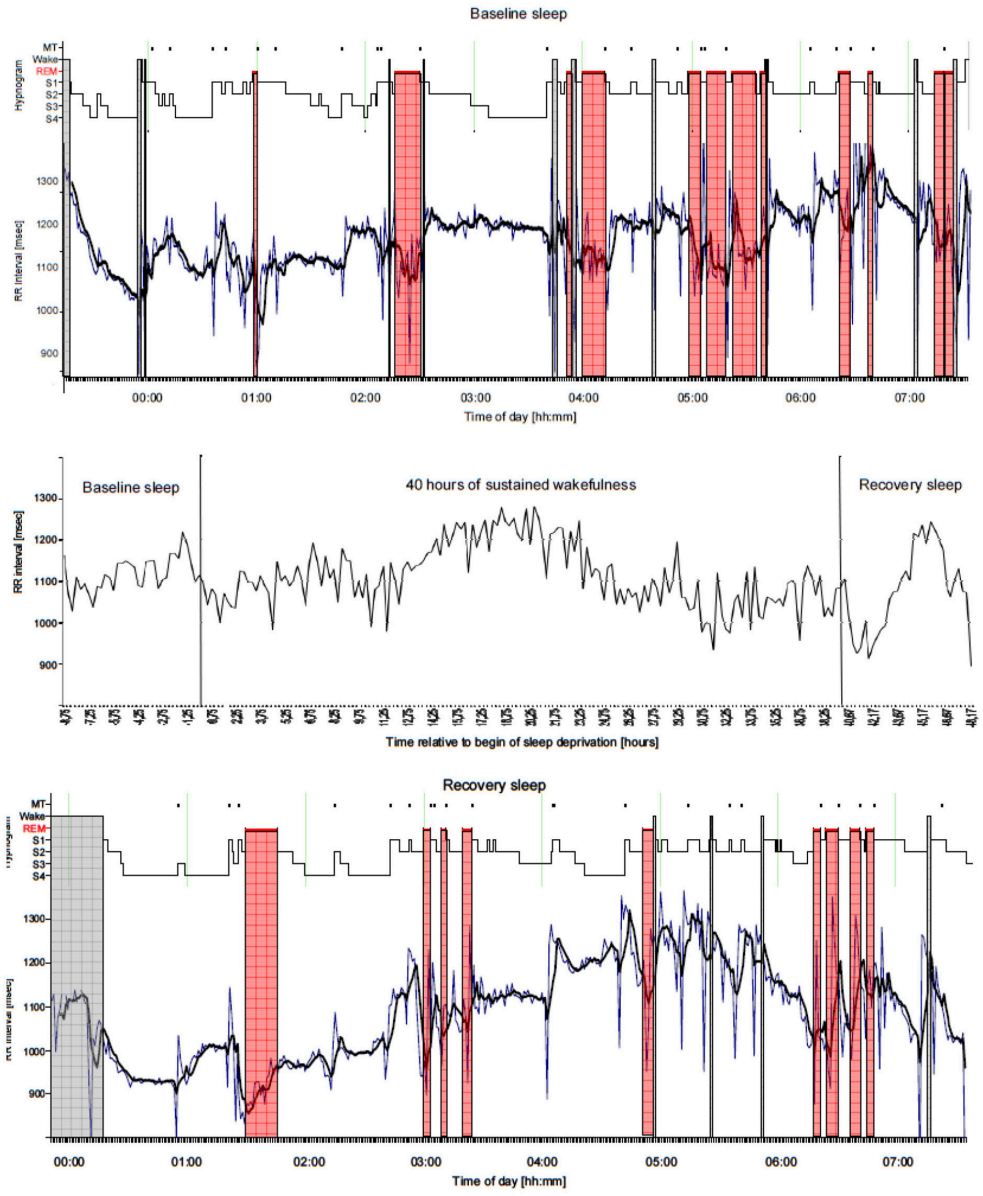
**Example of variation of heart rate (15-min mean) due to sleep stage, circadian timing, and sleep deprivation in a young male subject recorded for a period of altogether 56 h (middle panel)**. The recording consists of a Baseline sleep recording at night of 8 h, followed by a period of 40 h of sustained wakefulness inducing sleep deprivation, and finished with a Recovery sleep recording at night. For Baseline sleep and Recovery sleep periods 1-min mean and 5-min moving average values of heart rate are plotted and aligned to the sleep stage distribution (Hypnogram, 30 s resolution) in addition (upper and lower panel). It can be seen that heart rate is modulated mostly by sleep stages resulting in quite stable values during NREM sleep (sleep stages S1, S2, S3, S4) and highly variable values during REM sleep (marked with red color bars) as well as during wakefulness (marked with gray color bars). In addition changes in heart rate in part occur due to sleep stage changes accompanied by body movements (MT). In addition throughout sleep a global trend for longer RR intervals (→ lower heart rate) in the morning hours due to circadian modulation could be observed. During Recovery sleep this effect is more pronounced maybe because of the modified sleep profile due to the rebound character of sleep after 40 h of wakefulness. During the 40 h period of sustained wakefulness starting at 08:00 h in the morning and finishing in the late evening the following day it can be clearly seen that heart rate is modulated in a sinusoidal fashion by the circadian system. Although no sleep is present, the RR interval maximum (→ lowest heart rate) occurs in the morning hours after ~20 h of wakefulness. In addition fluctuations of heart rate can be observed throughout the entire 40 h period caused by a limited amount of activity and different cognitive tasks. MT, Movement Time; Wake, Wake stage; REM, Rapid Eye Movement sleep stage; S1, None REM (NREM) sleep stage 1; S2, NREM sleep stage 2; S3, NREM sleep stage 3; S4, NREM sleep stage 4.

Sleep stage associated changes in MSNA and in vagal nerve activity affect heart rate as well. Snyder's physiological investigations demonstrated that heart rate decreases during sleep and reaches lowest values during deep sleep (Snyder et al., 1964). Correspondingly during deep/slow wave sleep MSNA has very low levels. The parasympathetic nervous system dominates (Somers et al., 1993). During deep sleep, physical recovery occurs, and basal metabolic rate falls to its lowest level as well. Consequently, mean heart rate and HRV are maximally reduced. In REM sleep, in contrast, brain activity returns to a high level and the cortex is busy with the processing of mental activities. The autonomic system is likewise activated, and high levels of varying sympathetic tone are encountered (Somers et al., 1993). The mean heart rate is again higher, with values similar to those in light sleep, almost as high as during relaxed wakefulness. HRV is elevated in addition. Pronounced variation in heart rate is apparent, without association to physical load—still during sleep. In addition to these sleep stage associated influences, heart rate and HRV are subject to circadian modulation. They are further influenced by behavior prior to the sleep period, for example by extended wakefulness periods (i.e., sleep deprivation experiment with increased sleep pressure; Glos et al., 2014). Figure 3 show an example illustrating variation in heart rate caused by sleep stage, circadian timing, and by 40 h wakefulness/sleep deprivation in one subject recorded continuously for a period of altogether 56 h.

### Non-linear analysis of long-term heart rate variability

Attempts to identify sleep apnea on the basis of heart rate, and with computer-aided techniques, have encountered the problems described above with classical frequency-analysis procedures. This has led to consideration and development of new computational methods taken from statistical physics. These techniques were previously applied to the analysis of weather data, water-level information, and stock-exchange price fluctuations, and were widely considered to be methods of “chaos analysis.” The objective of these methods in general is to analyze data that appear random and to detect an inner structure and patterns of order that deviate from pure random behavior and that demonstrate phenomena of determinism, otherwise termed as “deterministic chaos.” One primary attempt here is to analyze the extent to which one value (of heart rate) depends on an immediately preceding value (of heart rate). If there is strong dependence between two subsequent heart beats, the relationship is termed correlated behavior. If there is random dependence between two heart beats, then the relationship is termed uncorrelated behavior.

The analytical procedures applied here are included among the methods of non-linear dynamics—e.g., an evaluation of complexity. In this way, investigation using the methods of non-linear dynamics has taken place for HRV, throughout an entire night, with patients with sleep apnea (Ivanov et al., 1996). The method used here analyzes beat-to-beat variability applying detrended fluctuation analysis (DFA). Although differences have been found between patients with sleep apnea and healthy test subjects, these disparities have not sufficed to establish distinct differentiation in terms of medical diagnosis.

One difficulty in the employment of DFA entails sudden fluctuations in dynamics: as they take place, for example, in weather data and in economic data series. Such alterations render it impossible to find uniform patterns of behavior for beat-to-beat variability in heart rate. We have observed major disturbances in heart rate with changes in sleepers' body positions at night, and during transition from one sleep stage to the next. To improve the analysis, therefore, the time course of nocturnal heart rate was broken down according to the various sleep stages, and the local disturbances resulting from stage transitions were discarded (Bunde et al., 2000). In other words, sequences of “pure” sleep stages were prepared for studying of HRV with DFA. Only as a second step, beat-to-beat sequences of heart rate were once again investigated for variability and sleep apnea. Investigation was performed to examine the extent to which one heartbeat interval is correlated with the subsequent heartbeat interval. It then became apparent that systematic investigation of HRV—on the basis of pure sleep-stage episodes from which the transition phases had been disregarded—revealed distinct and highly pronounced differences between the sleep stages. These differences were found in the beat-to-beat regulation of heart rate. In deep/slow wave sleep, there is a virtually uncorrelated behavior pattern from beat to beat, whereas extensively correlated behavior exists from beat to beat during REM sleep. These differences were greater between the various sleep stages than the differences found for episodes of heart rate with and without sleep apnea (Bunde et al., 2000).

These results were initially surprising, since the influence of sleep apnea on heart rate appears so pronounced and distinct. However, the marked alterations in sympathetic and vagal tone throughout the distinct stages of sleep provide a very good explanation here. Changes in sympathetic and vagal tone with respect to heart rate are evidently not so distinctly visible, since they are smaller in amplitude. They, however, are highly apparent in the beat-to-beat variation in heart frequency. This also explains why the differences between the sleep stages as determined by classical frequency analysis could in fact be determined, yet why they were not greatly pronounced. Indeed: in frequency analysis, not only the frequencies are taken into account, but precisely also the amplitudes at the respective frequencies—i.e., the so-called spectral power. Investigations of heart rate during sleep, furthermore, disclosed that influences of autonomic nervous system activity on heart-rate regulation are so dominant that they still prevail during sleep apnea and that they also allow distinction between sleep stages among these patients as well (Bunde et al., 2000). With respect to beat-to-beat variability, the cyclical variation in heart rate caused by sleep apnea therefore signifies merely a relatively minor additional disturbance. The results accordingly suggested the feasibility of a new procedure for determining differences between sleep stages (Penzel et al., 2003). The greatest differences arise between deep sleep on the one hand—with virtually uncorrelated beat-to-beat regulation—and REM sleep on the other, with extensively correlated beat-to-beat regulation of heart rate.

### Analysis of short-term heart rate variability

Short-term HRV and complexity have also been studied for many years. Early works include a test of several short-term measures of HRV to predict myocardial infarction from day and night-time recordings (Bigger et al., 1993). Related to our subject, a study of alternations of short-term HRV by obstructive sleep apnea was previously presented (Narkiewicz et al., 1998). Analyzing the increments between successive heartbeat intervals, short-term anti-correlations between the increments' signs (i.e., between accelerations and decelerations) have been identified (Kantelhardt et al., 2002). These anti-correlations were strong during deep sleep/slow-wave-sleep, weaker during light sleep, and even weaker during REM sleep, a finding very useful for modeling transient correlations in heartbeat dynamics during sleep considering the sleep stages (Kantelhardt et al., 2003). This has very recently been used as a starting point for a more sophisticated model (Soliński et al., 2016) with program code available on PHYSIONET (Goldberger et al., 2000).

Deceleration capacity (DC), which describes how quickly the heart rate decelerates within two beats, is lower during REM sleep and deep sleep as compared to light sleep and wakefulness (Schumann et al., 2010). This specific parameter decreases with age. We note that low values of DC have previously been shown to predict an increased mortality after myocardial infarction (Bauer et al., 2006). More recently, a complexity analysis based on conditional entropy supported the interpretation that REM sleep is a relatively high risk period compared to non-REM stages when aging (Viola et al., 2011).

Heart rate variability can be analyzed through various techniques such as those proposed in the seminal paper from the Task Force (Task force of the European Society of Cardiology and the North American Society of Pacing and Electrophysiology, 1996). These are mostly linear methods based in the time and frequency domain. Time domain parameters are calculated on the basis of the RR-intervals using simple statistical methods. While the mean heart rate is the simplest, the standard deviation over the whole time series (sdNN) is the most prominent measure used to describe what is considered HRV. However, none of the existing methodologies proved to be reliable in describing physiology and pathophysiology of cardiovascular regulation. We developed appropriate models by applying sophisticated methods to data of subjects with different pathologies. These models include many parameters from non-linear dynamics, a necessity based on insights such as those from a study of the complexity of the sinus node activity modulation system. Examples include methods based on symbolic dynamics, renormalized entropy, finite time growth rates, recurrence quantification analysis, large-scale dimension densities (Wessel et al., 2007).

The lack of widespread clinical use of these methods 20 years after the Task Force publication even despite much statistical data suggesting the predictive power of various cardiovascular indexes indicates a need for a new approach to clinical applicability. More clinical studies using these parameters are clearly needed, possibly focusing more on the question of additional information contained in existing indexes, than the development of new parameters.

Following these thoughts, we performed studies including respiration as a cofactor. Starting with asking “Is the normal heart rate “chaotic” due to respiration?” (Wessel et al., 2009), we demonstrated the influence of respiration on short-term HRV recordings. A recent case report (Sidorenko et al., 2016) presented a prominent case of this effect. In this case power spectra of HRV and respiration demonstrated an almost identical picture. A sudden change of the respiratory pattern caused a shift of the power distribution function toward lower frequencies. As a result the power in the LF band increased. This effect can lead to wrongly interpreted information when focusing on power in spectral bands. This potential error can be avoided by simply recording respiration in addition to heart rate and considering this effect when interpreting HRV power bands. Even if it is not possible to record respiration directly using respiratory sensors, the respiratory signal can be estimated with considerable accuracy regarding respiratory rate from the electrocardiogram itself—see below. Complex signal processing methods such as cardiorespiratory synchronization and coordination are providing further potential improvements for clinical applications.

## Respiration-related changes of heart rate and ECG

### Respiratory sinus arrhythmia and the baroreflex

**Figure 4:**
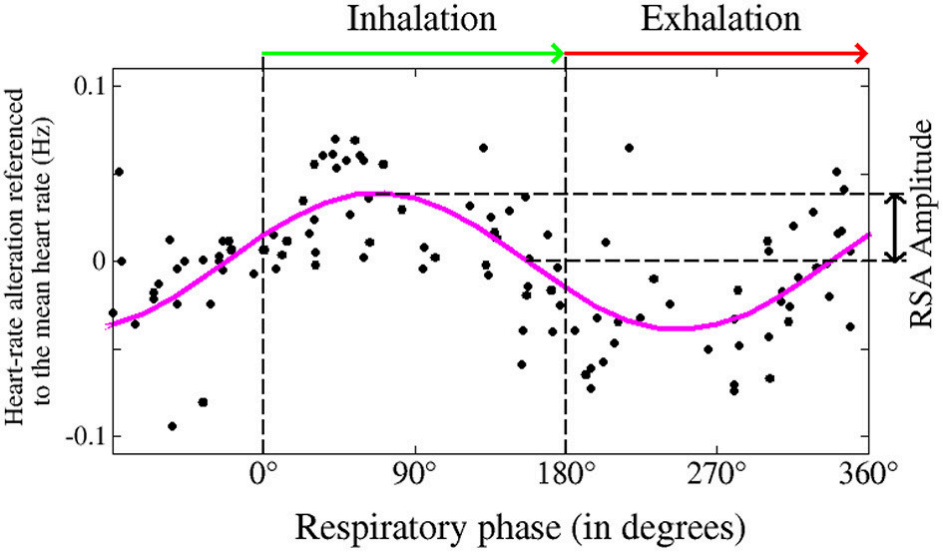
**Occurrences of heartbeats (•), as a function of the respiratory phase (0° for initiation of inhalation; 180° for beginning of exhalation), as taken from a typical subject during deep sleep**. The heart-rate trend (magenta plot) shows respiratory sinus arrhythmia (RSA)—accelerated heartbeat during inhalation and retarded heartbeat during exhalation. The intensity of the RSA is determined by the amplitude of sinus modulation.

The regulation of breathing and heartbeat are intimately coupled, as has been known for many years from basic physiology investigations (Koepchen and Thurau, 1959; Moser et al., 1995). The best studied mechanism of this cardiopulmonary coupling (CPC) is respiratory sinus arrhythmia, which also constitutes a major share of HRV. Respiratory sinus arrhythmia describes the respiratory-gated fluctuation of the heart rate: during inspiration, the heart rate increases; during expiration, it decreases again. Petr Einbrodt, in 1860, was the first to describe this coupling. Components which contribute to this regulation are MSNA, vagal nerve activity, respiratory timing, and pulmonary stretch receptors (Stoohs and Guilleminault, 1991; St. Croix et al., 1999; see Figure 4 for an example). In daytime during exercise, respiratory sinus arrhythmia is usually not visible, or is so weak that it cannot be depicted. During relaxation at rest and during sleep, in contrast, it is much larger and easily recognizable (Raschke, 1987). Early studies were even able to describe correlation between the extent of coupling and the various sleep stages. So the respiratory sinus-arrhythmia is significantly smaller in REM sleep than in Non-REM sleep (Bartsch et al., 2012).

Some studies demonstrated that respiratory sinus arrhythmia is not only the direct effect of respiration on heart rate but also the response of heart rate to the respiratory modulations of arterial pressure mediated by baroreflex (Eckberg, 2009). Therefore, two different simple models, the respiratory gate (Eckberg, 2003) and the deBoer-Karemaker-Strackee-Model (de Boer et al., 1985) were developed in order to explain this phenomenon. Recently it was suggested that more complex models should be utilized to describe cardiorespiratory coupling (Riedl et al., 2010; Porta et al., 2012; Runge et al., 2015).

### Recognition of sleep apnea with heart rate and morphology of the ECG

In 2000 at the Computers in Cardiology Conference, in Boston, USA, a public competition was inaugurated as part of a congress held by engineers of biomedical technology who are professionally involved with ECG analysis. This competition focused on solving a problem encountered in ECG analysis: recognition of obstructive sleep apnea by study of nocturnal ECGs (Penzel et al., 2002). ECGs from healthy test subjects, patients with moderate sleep apnea (up to 100 min of apnea per night), and patients with pronounced sleep apnea (more than 100 min with sleep apnea per night) were made available on a file server of PHYSIONET (Goldberger et al., 2000). A total of 35 ECG recordings were provided for training purposes, and 35 recordings were provided for purposes of analysis to competition participants. Additional respiratory signals were provided for some subjects in order to illustrate the underlying mechanisms of apnea to be studied. Out of 12 competitors two teams succeeded in correctly classifying all 35 subjects into the three groups, and were even able to correctly identify minutes with or without sleep apnea events in 92 and 94% of all 17,268 min of recordings presented (Penzel et al., 2002).

What made the two teams better than the other teams? In addition to data on the cyclical variation in heart rate, these teams had also analyzed the corresponding ECG plots (Penzel et al., 2002). This indeed revealed that respiration modulates the R wave and the T wave of ECGs in their amplitudes due to the movement of the cardiac electric axis imposed by respiration. This respiratory component is predominantly mechanical due to shifting of the heart with each breath, which each intrathoracic pressure change (see next section). This phenomenon had been known for long, but had not been exploited in this context (Moody et al., 1986). The so-called electrocardiographically derived respiration signal (EDR)—i.e., data on respiration as derived from an ECG—enables evaluation of the respiration activity and, in turn, detection of apnea and hypopnea events. Although when employed alone, EDR also demonstrates weaknesses in recognition of apnea and hypopnea events, it offers—in combination with information derived from cyclical variation in heart rate—an astonishingly high level of certainty in detecting sleep-related breathing disorders from ECGs. We note that respiration can also deform the R- and T-waves and thus affect the duration of heartbeat intervals as well, so that rhythmical modifications of amplitude and intervals are not completely independent (Lombardi et al., 1996; Porta et al., 1998).

### Reasons for morphology alterations of the ECG during sleep apnea

The certainty of EDR-based detection of sleep-related breathing disorders arises from the fact that the influence of breathing on an ECG is mainly mechanical in nature, and is therefore mainly independent of factors that affect the cyclical variation in heart rate. As a result, the combination of evaluation of autonomic influences on heart rate and assessment of the mechanical influences on the ECG (with EDR) enables good detection of apnea events. If further factors are included—oxygen saturation, snoring, and body movements—this enables application of an out-of-center sleep apnea recording system that can achieve a high degree of sensitivity and specificity for detection of sleep-related breathing disorders: without direct recording of respiration (de Chazal et al., 2009). This paves the way for reduced systems for sleep apnea detection.

In future, new systems that implement this combination or recording methods need to be validated. According to the classification of out-of-center sleep apnea diagnostic systems, these methods, which only rely on one recorded signal, the ECG, are not eligible for reimbursement by health care providers (Collop et al., 2011; Qaseem et al., 2014). Therefore, specific validation studies are needed to confirm sensitivity, specificity, and reliability of out-of-center sleep apnea recording systems, preferably in combination with oxygen saturation recording. Depending on just one recorded signal these systems have no backup in case of loose electrodes or signal failure, which is an obvious limitation.

## Cardiopulmonary coupling and synchronization

### Cardio-respiratory phase synchronization

**Figure 5:**
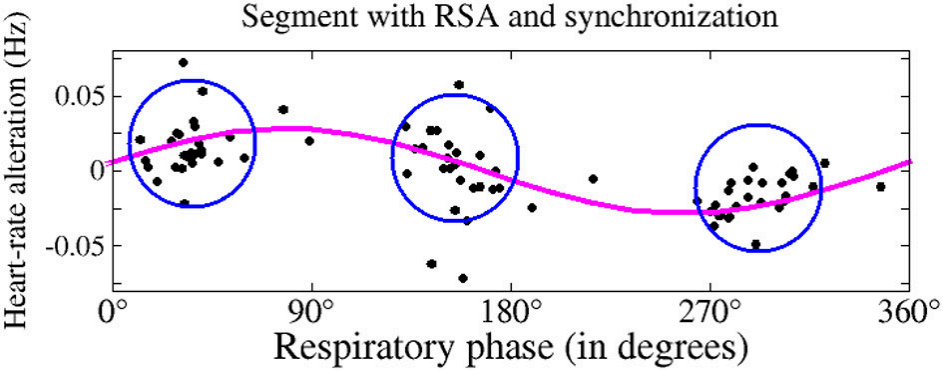
**This figure shows simultaneous occurrence of respiratory sinus arrhythmia (magenta plot) and cardiorespiratory phase synchronization (blue circles)**. During synchronization, heartbeats occur more frequently during particular respiratory phases: here, three heartbeats take place during one respiratory cycle (dots in the blue circles; based on Bartsch et al., 2012).

An important coupling phenomenon between systems is called phase synchronization. This had been described for the first time in the seventeenth century in conjunction with pendulum clocks (Huygens, 1673; Pikovsky et al., 2001). During cardiorespiratory phase synchronization, heartbeats occur more often during some phases of the respiratory cycle: e.g., at the beginning of the inspiration, at the end of inspiration, and in the middle of expiration (Figure 5; Schäfer et al., 1998, 1999; Toledo et al., 2002; Bartsch et al., 2007, 2012). The occurrence of cardiorespiratory phase synchronization is intermittent and not constant. This means that the phenomenon can be observed during a few percent of observational time only. For reliable tracking of phase synchronization throughout the night, surrogate data techniques are needed to check statistical significance for each detected synchronized episode (see, e.g., Toledo et al., 2002; Bartsch et al., 2007).

**Figure 6:**
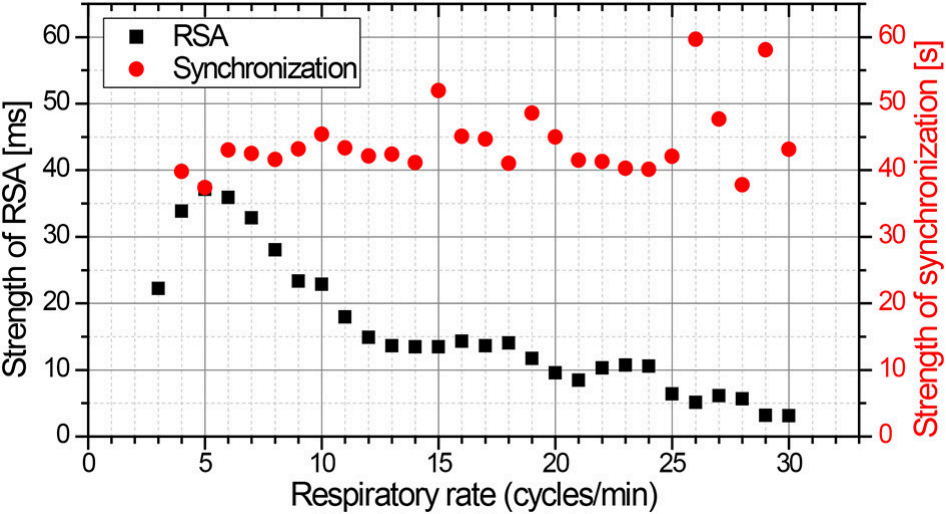
**The amplitude of respiratory sinus arrhythmia is distinctly a function of respiratory rate, and is most pronounced at ~5 respiratory cycles per minute (black squares)**. In contrast, the mean duration of episodes with phase synchronization exhibits no dependency on respiratory rate (red circles; based on Bartsch et al., 2012).

Phase synchronization between heart rate and respiration can occur independent of respiratory sinus arrhythmia. This is shown in Figures 4, 5. In addition, both coupling mechanisms are influenced by different physiological parameters. An important example for this influence is respiratory frequency. Whereas, the extent of respiratory sinus arrhythmia is distinctly dependent on respiratory frequency, this is not the case for phase synchronization (Figure 6; Bartsch et al., 2012).

The physical training condition of the persons examined is evidently significant for the extent of cardiorespiratory phase synchronization (Schäfer et al., 1998, 1999). Athletes have demonstrated pronounced synchronization between respiration and heartbeat, which leads to the conclusion that the occurrence of this synchronization represents ergonomically effective regulation. The influence of the extent and effectiveness of this coupling on physical or mental performance have not been determined until now. It is assumed that coupling could represent a good surrogate parameter for recovery periods after physical exercise.

More studies have systematically investigated this phase synchronization during sleep. This had been done for healthy subjects and also for patients with sleep-apnea by multiple groups (Cysarz et al., 2004; Kabir et al., 2010; Bartsch et al., 2012; Müller et al., 2012, 2014; Riedl et al., 2014; Solà-Soler et al., 2015). In healthy persons, it had been proven that the percentage of time spent with strong synchronization depends on sleep stages. Synchronization between respiration and heartbeat is observed for the largest percentage of time during deep sleep. In contrast to this the percentage of time with synchronization is smallest during REM sleep (Bartsch et al., 2012). This sleep-stage dependency is many times greater for phase synchronization than for respiratory sinus arrhythmia, and is likewise much greater than the variations in mean heart rate, HRV, and respiratory rate (Table 1). In patients with sleep disordered breathing, such as sleep-apnea, the time spent with synchronization is strongly impaired.

**Table 1:** **Relative changes of parameters during the various stages of sleep**.

**Parameter**	**Wakefulness**	**Light sleep (N1 and N2)**	**Deep sleep (N3)**
Heart rate	1.06	0.98	0.99
Heart rate variability (HRV)	1.15	0.97	0.93
Respiratory rate	1.02	0.95	0.92
Strength of RSA	0.90	1.33	1.37
Strength of Synchronization	1.6	2.7	4.2

The mean values for each sleep phase have been normalized to the corresponding value in REM sleep. While mean heart rate, HRV, and respiratory rate increase during wakefulness as compared with REM sleep, they drop during non-REM sleep (light and deep sleep). The opposite dependence is observed for the strength of respiratory sinus arrhythmia (RSA). In contrast, the strength of cardio-respiratory synchronization is weakest during REM sleep. Furthermore, a greater sensitivity with respect to transitions between stages of sleep is seen for synchronization—greater by a factor of ten than for RSA. This demonstrates that sleep regulation influences both aspects of cardiopulmonary coupling in different ways.

Another technique to evaluate cardiopulmonary coupling during sleep solely utilizes continuous ECG signals and derives the HRV (e.g., RR interval time series) as well as fluctuations in R-wave amplitude induced by respiration (Thomas et al., 2005). From the R-wave amplitude fluctuations, ECG-derived respiratory signal (EDR) is obtained again. The cross spectral power and coherence of the RR time series and corresponding EDR series are calculated for consecutive windows and the product of coherence and cross-spectral power is used to obtain the ratio of coherent cross power in the low-frequency (0.01–0.1 Hz) band to that in the high-frequency (0.1–0.4 Hz) band. Developing and applying this technique, Thomas et al. investigated recordings of cardiorespiratory polysomnography of healthy subjects and found that consolidated, stable non-REM sleep is characterized by high frequency coupling (0.1–0.4 Hz), increased absolute and relative delta power (Thomas et al., 2014), stable breathing and oxygenation, absence of arousals and blood pressure dipping. In contrast, unstable non-REM sleep is characterized by low frequency coupling (0.01–0.1 Hz), intermittent arousals, and non-dipping of blood pressure. In patients with obstructive and central sleep apnea, ventilation, and oxygenation abnormalities are observed (Thomas et al., 2005). An example of cardiopulmonary coupling in a patient with heart failure and Cheyne-Stokes breathing is depicted in **Figure 8**. Patients with heart failure have more often central sleep apnea than obstructive sleep apnea.

### Cardio-respiratory coordination

Cardio-respiratory coordination can be calculated by an analysis of both, the cardiac and the respiratory cycle. The coordination itself defines the mutual influence of the onsets of cardiac and respiratory cycles on each other (Moser et al., 1995). In particular cardio-respiratory coordination leads to a constant-time relationship between the cardiac and the respiratory cycle. Formally, it differs from the previously described phase synchronization by considering the alignments in the time domain instead of the phase domain and the bidirectional aspect. The cardio-respiratory coordination is mostly observed during anesthesia (Galletly and Larsen, 1997), rest (Raschke, 1991), sleep (Raschke, 1991; Cysarz et al., 2004), and during relaxed condition, similar as the cardio-respiratory phase synchronization. An indicator of the difference between both the phenomena is their different dependencies on sleep stages where cardiorespiratory coordination is most frequently detected in light sleep (Raschke, 1991) in contrast to the phase synchronization which has its maximum during deep sleep (Bartsch et al., 2012). Because of the prevailing observation during relaxed conditions, it has been assumed that disturbances like stress strongly reduce coordination as well as synchronization. One such example is obstructive sleep-apnea (Raschke, 1987; Kabir et al., 2010; Solà-Soler et al., 2015) and another is mental stress (Niizeki and Saitoh, 2012). As soon as a series of sleep apnea events occur, respiratory regulation follows the circulatory system. As a result, the coupling, the relationship between the two systems, is impaired. The impairment can be so strong that the coupling is no longer detected.

**Figure 7:**
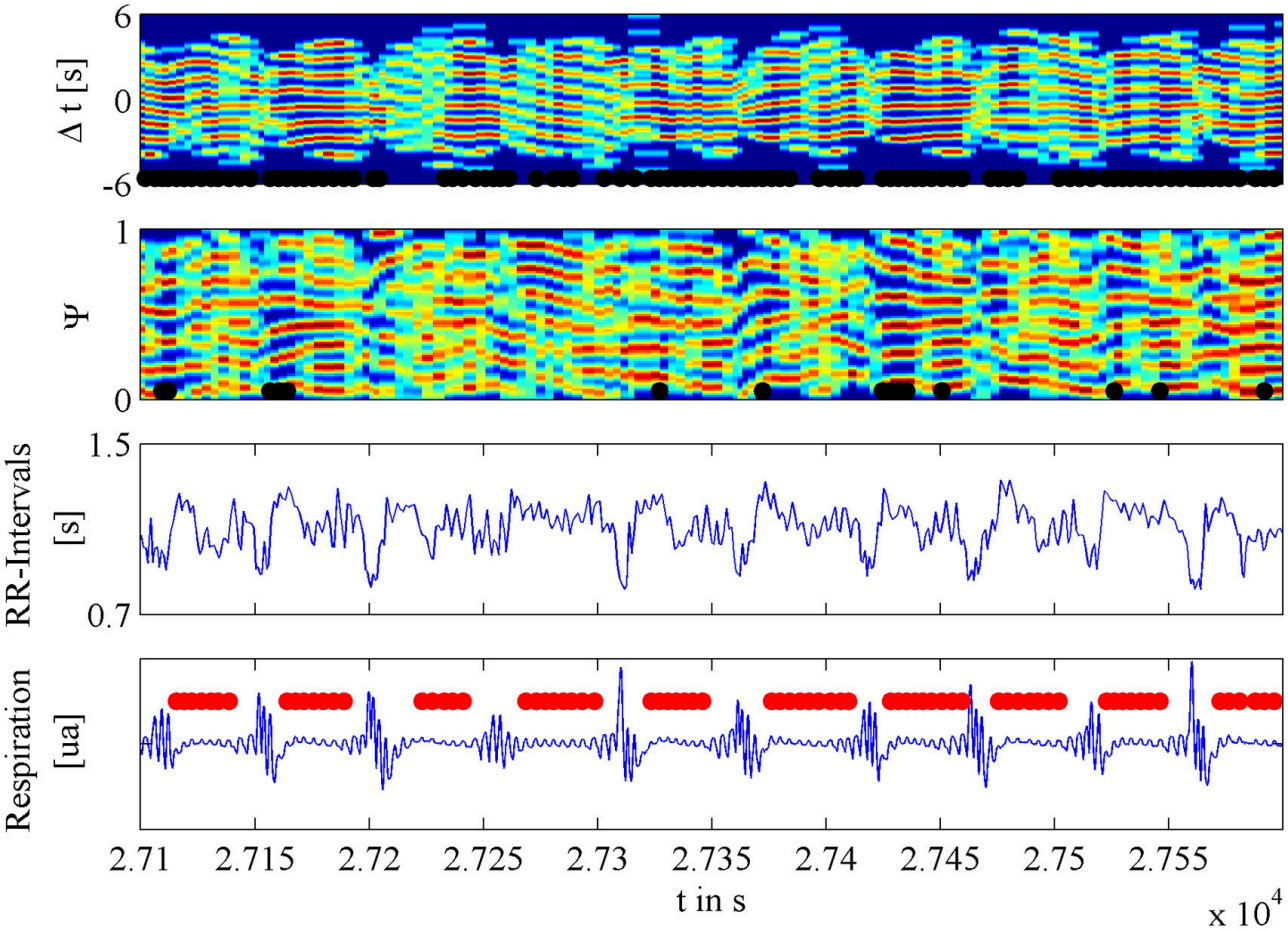
**This figure shows examples of cardiorespiratory coordination during and after apnea events**. The upper plot shows a so-called coordigram where the cardiorespiratory coordination is characterized by red horizontal strips. The black bars below display the detected episodes of coordination. For comparison, the second color-coded plot is the related synchrogram where again red horizontal strips characterize cardiorespiratory phase synchronization. As in the plot above, the black bars at the bottom display the detected periods of phase synchronization. The third plot shows the associated alterations of beat-to-beat intervals, with the characteristic cyclical variations that are triggered by apnea events. The fourth plot shows the associated signal for abdominal respiratory movement, with the characteristic pattern of consecutive obstructive apnea events (marked by horizontal red bars).

A recent analysis with much improved time resolution indicated that coordination takes place during apnea phases and is not extinguished by the brief hyperventilation phases that follow each single apnea event (Riedl et al., 2014; Figure 7). Coupling and coordination of respiration and the circulatory system are therefore considered as additional markers for cardiovascular regulation, similar to HRV (Togo and Takahashi, 2009; Tobaldini et al., 2013), blood pressure variability (Parati et al., 1995), and baroreceptor sensitivity (Parati et al., 2000).

Beside this, discussions regarding the origin of the cardio-respiratory coupling and the dominant direction of this mutual interaction are needed. This may require more basic physiological experiments.

## Discussion

New attempts by engineers and start-up enterprises are currently undertaken to develop cost-effective equipment on the basis of easily accessible devices, such as Smartphones, to record heart-rate and pulse-rate (Behar et al., 2013). Based on data gathered by Smartphone applications, spectral analysis and procedures involving nonlinear dynamic analysis are used to calculate HRV. Attempts are likewise being made to apply findings from the above-described studies to work with the signals involved. Other endeavors with Smartphone applications have attempted by means of simply recorded signals to detect sleep, to distinguish sleep stages, and also the occurrence of sleep apnea.

These applications are available at low cost and have found extensive use in the public. They are popular because insights into sleep and possibly sleep disorders can be obtained using a recording and an analysis conducted at home without consulting a sleep medicine center which would be expensive and would have considerable waiting time for an investigation. In contrast to cardiorespiratory polysomnography, none of these algorithms has been validated in appropriate clinical studies. Clarification is still required to determine the extent to which these possibilities offer diagnostic potential, or to which they merely increase individual concern and worry if something unusual is found. This is an example of a fuzzy transition from medical and physiological expertise to general knowledge and wellness applications in the general frame of quantify yourself.

Even if many cardiorespiratory polysomnography recordings are performed every day, i.e., in Germany between 1000 and 2000 each day, there is no validated processing or analysis for the one-channel ECG recorded with each of them—except for a simple descriptive statistics on heart rate values such as mean, maximum, and minimum values for the recording period. The reason for this low level analysis is the lack of automatic detection of irregularities/arrhythmias in a single-channel ECG, which are usually recognized only by visual inspection by a trained clinician. The reduced evaluation of ECG does not reflect methods and knowledge on HRV available up to date. Even established algorithms as well as new analysis algorithms for HRV and for cardiorespiratory coupling are not applied in sleep studies until today. With respect to HRV analysis this may be due to the interference between respiration and HRV parameters. Only controlled conditions in terms of paced breathing would allow a comparison of inter and intra individual changes. Cardiorespiratory coupling however does influence both interdependent systems and therefore can be very useful for clinical interpretation in polysomnography recordings. In order to promote this additional interpretation a closer interaction between clinicians and researchers on this topic is needed. This cooperation may help interpreting the so called autonomous arousal. The autonomous arousal describes changes in parameters as observed during autonomous nervous system activations which are not seen in parallel in EEG leads as central nervous activations. Instead they are short alterations in ECG and heart rate as well as blood pressure, often as a consequence of brief movements, respiratory irregularities such as sights or other phenomena observed during sleep. These brief events are not yet further evaluated and may contribute as sleep disturbing factors. Similar to respiratory related arousals (so called RERAs), clinicians observe movement and cortical activity and ECG related arousals (Fietze et al., 1999). These short transient events may be important for a differential diagnosis and furthermore for a decision in treatment of persons with reported sleep disorders.

New methods for the evaluation of ECG and blood pressure will allow better to distinguish between subjects being healthy or disturbed sleepers, as already seen in subjects with obstructive snoring, subjects with bruxism, or subjects with periodic leg movements without cortical arousals. These people do suffer without obvious indications in conventional sleep EEG parameters.

This uncertainty is especially significant in light of the necessary assumption that this form of investigation is being conducted not only with healthy subjects and with patients definitely suffering from sleep apnea, but also with persons who merely snore and others who represent transition forms between snoring and sleep apnea. The quality of such applications becomes particularly evident in the context of such borderline cases: which are even more widespread than cases of unequivocal sleep apnea itself. For this reason, reliable validation of such applications are required before diagnostic employment. Otherwise, they represent merely one more general application intended to measure data from individuals and to expand their personal digital environment, without sustainable background and without the possibility of specific and well-founded intervention.

**Figure 8:**
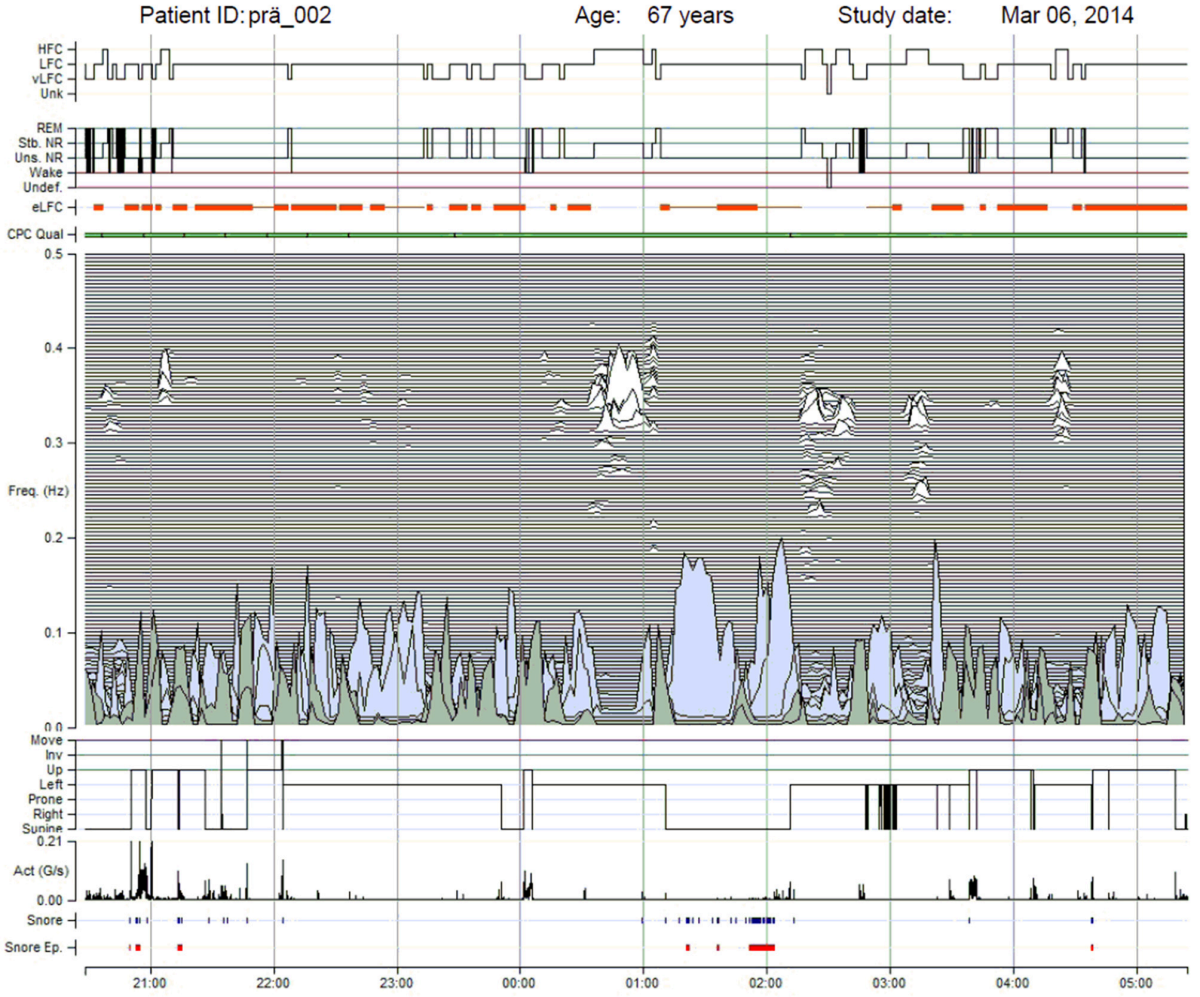
**This figure depicts the spectrogram of heart rate for a patient with heart failure and Cheyne-Stokes breathing**. The study took place in 2014 at the Interdisciplinary Sleep Medicine Center, Charité Universitätsmedizin Berlin, with application of the M1 device that records ECG, heart rate, respiration calculated by EDR, snoring, and body position. The plot results from an analysis of heart-rate variability: top signal, with marking corresponding to the dominance of high-frequency coupling (HFC), low-frequency coupling (LFC), and very low-frequency coupling (vLFC). The figure also shows an approximation of sleep stages: second signal block from the top with marking of REM, stable non-REM (shown as Stb. NR), unstable non-REM (shown as Uns. NR), and wake stages (shown as Wake). The plots at the bottom show the spectrograms of cardiorespiratory coupling (see main text), indicators for body position (up for upright, left, prone, right, and supine), the intensity of actigraphy (Act), and snoring events (Snore). This evaluation distinctly reveals impaired sleep.

The devices that are now commercially available and that address these concepts include, for example, a diagnostic early-warning instrument for characterization of sleep quality and assessment of sleep disordered breathing: the M1 SleepImage recording system (MyCardio LLC, Broomfield, CO, USA). The device is attached to the thorax with two self-adhesive electrodes. Then it records a single-channel ECG. In parallel it records snoring by means of a microphone, and body position and activity by an acceleration sensor. From the ECG the system determines the HRV and the EDR. With this the software calculates the degree of cardiopulmonary coupling (CPC) as described above (Thomas et al., 2005). The CPC can be depicted by a spectrogram (Figure 8). This helps to identify the frequency band in which CPC is present. Of particular interest for clinical applications are sleep disordered breathing such as sleep apnea. During sleep disordered breathing, CPC is elevated in the low frequency band, i.e., low-frequency coupling (LFC). It is furthermore possible to characterize existing sleep disordered breathing on the basis of various patterns in LFC. An initial study has identified greater likelihood of a narrow-band sample in LFC for central apnea events—whereas, in contrast, a more pronounced broad-band pattern is characteristic of obstructive sleep apnea (Schramm et al., 2014). This is of special interest, because the distinction between obstructive and central sleep apnea has diagnostic and possibly therapeutic consequences. Prospective studies are needed to verify these finding and to confirm results on large patient groups which are less preselected.

An additional possibility, likewise still in initial stages of investigation, would be the recognition of sleep-related breathing disorders among patients with cardiac pacemakers, by using data collected and provided by the pacemaker itself. In recent pacemakers (ICD and CRT devices), an impedance measurement can monitor breathing continuously and, in turn, evaluate sleep apnea and the severity of this disorder by calculating the number of apnea events per hour of sleep, just like regular diagnostic devices. It would be likewise necessary to investigate the diagnostic benefits of this integrated technique by means of clinical trials with large patient groups. Currently no treatment consequences are taken when apnea events are detected using this methodology.

## Summary

Analysis of ECG data and heart rate during sleep provides an appreciable diversity of information on the physiology and the pathophysiology of sleep-wake regulation. Assessment of nocturnal ECGs with respect to cyclical fluctuations of heart rate, combined with study of respiration-dependent alterations in ECG morphology (e.g., amplitudes of the R-waves and T-waves), allows reliable recognition of sleep-related breathing disorders. The quality of sleep itself can also be approximately evaluated by analysis of heart-rate variations. Deep sleep and REM sleep, to be sure, demonstrate characteristic properties in heart-rate variability.

Even now, new methods are being applied in practice by presenting sleep findings that already include analysis of sleep and sleep-related breathing disorders with the aid of long-term ECG systems, data from pacemaker ECGs, and information from innovative, reduced-scale recording systems. To arrive at solid diagnostic and therapeutic conclusions from these results, it will be necessary to conduct prospective validation studies and to perform clinical evaluation with parallel out-of-center sleep studies and polysomnography. In addition new algorithms are needed which allow an automated processing of heart rate and HRV which results in a conclusive report, similar to the report created from sleep stage scoring or respiration scoring.

## Author contributions

All authors equally contributed to write the review. The figures were prepared by different authors listed and using their specific algorithms. Discussions and perspectives were discussed with all authors.

## Funding

The project no. LQ1605 from the National Program of Sustainability II and FNUSA-ICRC (No. CZ.1.05/1.1.00/02.0123) supported this research. The Deutsche Herzstiftung supported CG for 1 year. JWK acknowledges support from the German Research Society (DFG, grant KA 1676/4) and the German Israeli Foundation (GIF, grant I-1298-415.13/2015).

### Conflict of interest statement

The authors declare that the research was conducted in the absence of any commercial or financial relationships that could be construed as a potential conflict of interest.
